# Recent advances in the pathogenesis and treatment of paroxysmal nocturnal hemoglobinuria

**DOI:** 10.12688/f1000research.7288.1

**Published:** 2016-02-23

**Authors:** Lucio Luzzatto

**Affiliations:** 1 University of Firenze, Piazza di San Marco, 4, Florence, 50121, Italy; 2 Department of Haematology, Muhimbili University Hospital, Dar es Salaam, Tanzania

**Keywords:** Paroxysmal nocturnal hemoglobinuria, hemolytic anemia, clonal disorder, PNH, GPI

## Abstract

Paroxysmal nocturnal hemoglobinuria (PNH) is a very rare disease that has been investigated for over one century and has revealed unique aspects of the pathogenesis and pathophysiology of a hemolytic anemia. PNH results from expansion of a clone of hematopoietic cells that, as a consequence of an inactivating mutation of the X-linked gene *PIG-A*, are deficient in glycosylphosphatidylinositol (GPI)-linked proteins: since these include the surface membrane complement-regulatory proteins CD55 and CD59, the red cells arising from this clone are exquisitely sensitive to lysis by activated complement. Until a decade ago, the treatment options for PNH were either supportive treatment – often including blood transfusion, anti-thrombosis prophylaxis, and sometimes thrombolytic therapy – or allogeneic bone marrow transplantation. Since 2007, PNH has received renewed and much wider attention because a new form of treatment has become available, namely complement blockade through the anti-C5 monoclonal antibody eculizumab. This brief review focuses on two specific aspects of PNH: (1) response to eculizumab, variability of response, and how this new agent has impacted favorably on the outlook and on the quality of life of patients; and (2) with respect to pathogenesis, new evidence supports the notion that expansion of the PNH clone results from T-cell-mediated auto-immune damage to hematopoietic stem cells, with the GPI molecule as target. Indeed, GPI-specific CD8+ T cells – which have been identified in PNH patients – would spare selectively GPI-negative stem cells, thus enabling them to re-populate the marrow of a patient who would otherwise have aplastic anemia.

## Introduction

Paroxysmal nocturnal hemoglobinuria (PNH) is a unique disorder in more ways than one ^1^. First, it is a hemolytic anemia, but, unlike any other hemolytic anemia, it is frequently associated with pancytopenia. Second, it is a thrombophilic condition in which it is not unusual for thrombosis to take place, paradoxically, in a thrombocytopenic patient. Third, although it is due to an intrinsic abnormality of the red cell, it is an acquired disorder ^2^. This last characteristic feature, a long time ago, led to the notion and then to the demonstration that PNH is a clonal disorder ^3^, and we now know that the clone originates from a hematopoietic stem cell (HSC) with a somatic mutation that inactivates the X-linked gene *PIG-A* ^4^. This gene encodes one of the subunits of a specific *N-*acetylglucosamine transferase ^5^, the first enzyme of the complex pathway that leads to the synthesis of the glycosylphosphatidylinositol (GPI) molecule that tethers many proteins to the cell surface ^6^. As a result, the cells belonging to the PNH clone are severely or totally deficient in these proteins: they have a GPI-negative phenotype. Since the two red cell surface complement regulators CD55 and CD59 are GPI-linked proteins ^7^, red cells belonging to a PNH clone are exquisitely sensitive to complement, and they will hemolyze when complement is activated: indeed, in most cases intravascular hemolysis is a dominant pathophysiological feature of PNH ^8^.

It has been made clear from several animal models that inactivating mutations of *PIG-A* do not confer to HSCs a selective growth advantage ^9, 10^. Indeed, to understand what enables the *PIG-A* mutant GPI-negative clone to expand has been a challenge. Three hypotheses have been considered: (i) since rare somatic mutations are present in every normal person, and since relatively few HSCs are active in normal hematopoiesis, one *PIG-A* mutant HSC – even though it has no selective advantage – might simply by chance (genetic drift) produce a large proportion of the peripheral blood cells, and these will be GPI negative ^11^; (ii) there is a close relationship between aplastic anemia (AA) and PNH, and it has been suggested that this is anything but a coincidence; the cell-mediated auto-immune attack that is believed to cause AA may spare selectively GPI-negative HSCs: in other words, the bone marrow environment prevailing in PNH patients creates for the *PIG-A* mutant GPI-negative clone a growth advantage which is not intrinsic but is conditional on the environment ^12^; (iii) in the *PIG-A* mutant clone, there may be additional mutation(s) that confer to the respective subclone(s) an intrinsic growth advantage. In two patients with PNH, a mutation of the *HMGA2* gene may have played this role ^13^, and *HMGA2* over-expression has been reported in additional cases ^14^. In addition, a recent massive parallel sequencing study, while confirming that the only gene mutated in all cases is *PIG-A*, has revealed mutations in several other genes, some of them already known to be mutated in myelodysplastic syndromes ^15^.

There are many published reviews on PNH and here we intend to focus only on selected recent developments.

## Novel therapy

The last decade has been marked by the clinical trials of eculizumab (ECU) ^16, 17^, followed in 2007 by the prompt introduction of this agent into standard therapy. ECU is a humanized monoclonal antibody specific for the human plasma complement component C5: by binding to C5, ECU blocks the distal complement pathway and thus protects PNH red cells from complement-mediated lysis. In patients with PNH, anemia *per se* causes fatigue; at the same time, intravascular hemolysis often entails unpleasant and sometimes severe symptoms, such as abdominal pain, dysphagia, and erectile dysfunction. ECU terminates intravascular hemolysis in virtually all patients: as a result, these symptoms are abrogated, and fatigue from anemia is often alleviated. Up to two-thirds of patients who were transfusion dependent become transfusion independent ^18^. It is not an exaggeration to say that ECU has changed the life of many PNH patients.

## The spectrum of response to eculizumab and pharmacogenetics

With respect to the response to ECU, we must consider at least two different end-points: (a) inhibition of intravascular hemolysis consequent on complement blockade and (b) overall clinical benefit. The cases where response to ECU fails to meet end-point (a) are very rare: they have been reported in patients from Japan who have a specific mutation in the *C5* gene, which entails in the C5 protein an Arg885His amino acid replacement, which in turn prevents binding of ECU ^19^. This mutation is polymorphic in Japan (heterozygote frequency of about 3.5%) and in China, but to date it has not been observed elsewhere.

**Figure 1.**
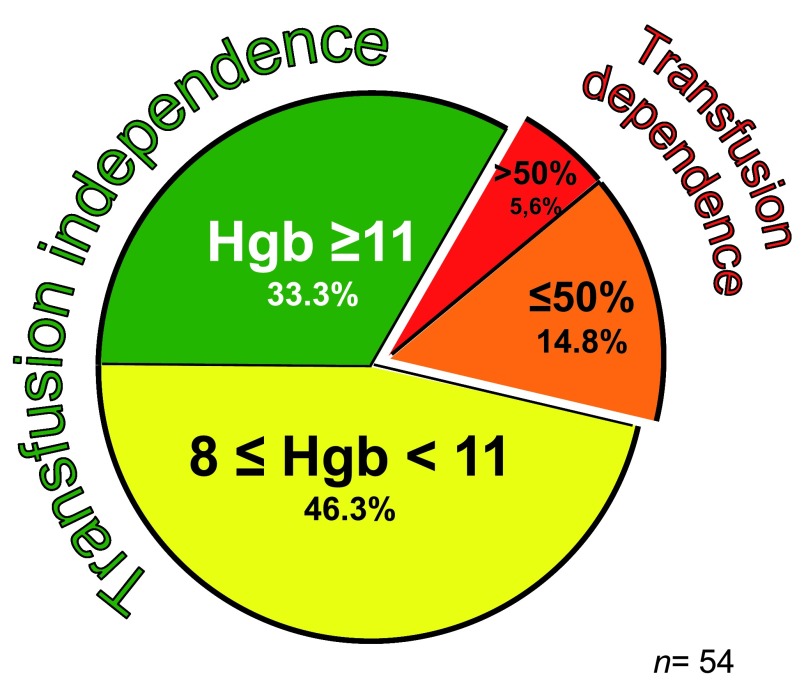
Variable hematological response to eculizumab treatment (modified from 18).

In contrast to (a), which behaves like an all-or-none end-point, clinical benefit (b) is a continuous variable. Of those patients who become transfusion independent, some experience a net increase in hemoglobin level as well (see Figure 1); others stay on more or less that same hemoglobin level to retain what they previously needed transfusion support for ^18^. Some patients (about 25% in our experience) still need recurrent blood transfusion (usually at less frequent intervals than before); however, even these patients report improved quality of life because they no longer experience the unpleasant subjective symptoms listed above.

**Figure 2.**
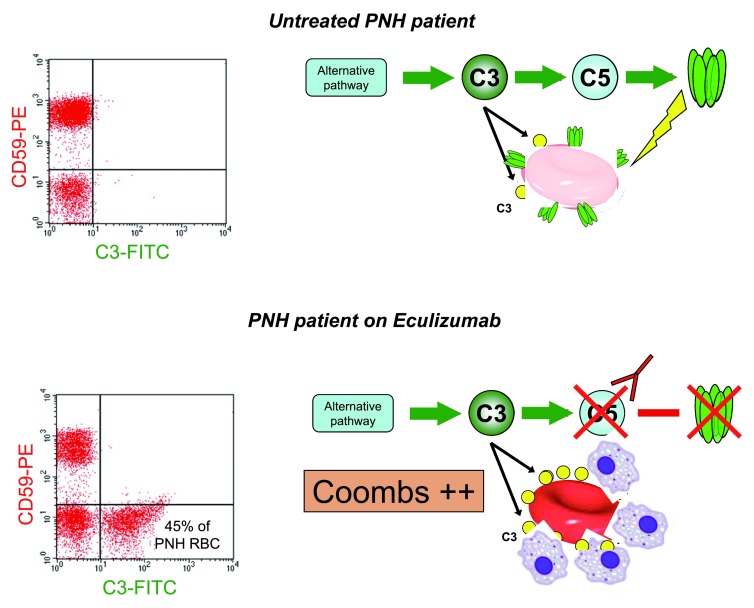
Mechanism of (iatrogenic) extravascular hemolysis in a paroxysmal nocturnal hemoglobinuria (PNH) patient on eculizumab. In untreated patients (upper cartoon), as soon as C3 (yellow circles) is bound to a GPI-negative red cell (lacking CD55), C5 will be activated, the membrane attack complex (MAC) will form, and the red cell (which also lacks CD59) will be lyzed: accordingly, no red cells with C3 bound are seen in the upper flow cytometry pattern on the left. In patients on eculizumab (lower cartoon), with C5 blocked, no MAC is formed and there is no lysis; in return, a much larger number of C3 molecules accumulate (see lower flow cytometry pattern on the left); thus, the red cell is opsonized and will be prey to macrophages. This is the likely mechanism of extravascular hemolysis in PNH patients on eculizumab (modified from 18).

This variability of response ( Figure 1) is not yet fully explained. In some patients, a poor or suboptimal response may indicate that the extent to which bone marrow failure was contributing to anemia had been under-estimated. In others, a suboptimal response is related at least in part to an important shift in pathophysiology that takes place in patients on ECU (see Figure 2). In essence, because GPI-negative (PNH) red cells are protected from complement lysis, they survive much longer in circulation (at first sight regarded as paradoxical, an increase in the proportion of PNH red cells is a typical feature of patients on ECU). ECU affects only the distal complement pathway, thus neutralizing the handicap caused by the deficiency of CD59 on PNH red cells, but since these are also deficient in CD55, there will be a gradual accumulation of C3 fragments on their surface (see Figure 2), and these C3-opsonized red cells will be susceptible to phagocytosis by macrophages in the reticuloendothelial system ^20, 21^. This mechanism of extravascular hemolysis – which we must regard as iatrogenic – causes the Coombs test to become positive ^22^ (it is classically negative in untreated PNH); more importantly, it may limit the improvement in anemia because it develops at the same time that intravascular hemolysis ceases.

At least one factor that influences markedly the development of this consequence of ECU treatment is, once again, genetic and complement related. When patients on ECU are stratified according to their genotype for the complement receptor gene *CR1*, it is seen that among those who have a suboptimal hematological response, homozygotes for the low-affinity allele *L* are markedly more common, and homozygotes for the high-affinity allele *H* much rarer than expected; heterozygotes are intermediate, indicating a dosage effect ^23^.

## Thrombosis in paroxysmal nocturnal hemoglobinuria

Venous thrombosis, particularly in abdominal veins or in intracranial veins, remains one of the most feared complications of PNH. Although the literature on this subject is vast, the mechanism that makes PNH the most vicious acquired thrombophilic state known to medicine ^18^ remains elusive. Thromboplastin-like substances from hemolyzed red cells, inappropriate platelet activation, and failure of fibrinolysis may all play a role ^24^. But in recent years the most significant change has been caused, also in this area, by the use of ECU. Although there has been no formal trial on the impact of ECU on thrombosis, patients on ECU have some changes in laboratory parameters of hemostasis ^25^, and they have fewer thrombotic episodes than one might have expected from previous experience ^26, 27^. This is obviously of great clinical importance; in addition, it indicates that complement blockade (whether it acts *via* red cells or in any other way) does protect from thrombosis, although not completely because thrombosis on ECU can still occur ^27^.

At any rate, the risk of thrombosis is still highly relevant to the management of PNH patients for several reasons. First, to many patients living in many parts of the world, ECU is not available, mainly because it is too expensive: for these patients, it is important to have a clear policy with respect to prophylaxis of thrombosis (for example, see 18). For these patients, it is likely that coumadin will be replaced soon by new oral anticoagulants (NOACs), although there is as yet little experience in PNH with these agents. Second, patients with PNH who also have an inherited thrombophilic state ( *e.g.* a prothrombin mutation or an anti-thrombin III defect) are at even greater risk than the others and one must consider, even when they are already on ECU, having them on anticoagulants as well. Third, patients not previously diagnosed with PNH may present with recent thrombosis: in these cases, it would seem reasonable to introduce ECU immediately, but if this is not possible one ought to consider thrombolytic therapy with tissue plasminogen activator (tPA), which can be highly effective ^28^. Fourth, in some PNH patients, a special problem, almost invariably secondary to splenic vein and/or portal vein thrombosis, is splenomegaly with hypersplenism, which may cause cytopenias or make them worse: in such cases, selective splenic artery embolization (SSAE), carried out in steps, will reduce spleen size and alleviate cytopenias ^29^. An extra bonus of this procedure is that when in a patient on ECU extravascular hemolysis is severe, it may be relieved by SSAE ^29, 30^, a safer alternative compared to splenectomy ^31^.

## The mechanism of clonal expansion

As mentioned in the introduction, in order to account for the expansion of a GPI-negative blood cell population in patients with PNH, one possible explanation is a selective environment. The selective process must be able to differentiate stem cells that are GPI positive from those that are GPI negative, even if they are otherwise undistinguishable ^32^. Cells of the immune system are specialized in recognizing chemical structures, and therefore they are good candidates for being the agents of highly sophisticated selection. The immune system is already presumed to be involved in the pathogenesis of idiopathic AA ^33^, and although the phrase AA-PNH syndrome was coined perhaps with the idea that this was a peculiar subtype of PNH ^34^, in our experience and in that of others ^35^, a history of more or less severe AA at the time of diagnosing PNH is present in at least one-half of all cases. It may perhaps soon become the rule rather than the exception. This is consistent with the notion that expansion of a GPI-negative clone, characteristic of PNH, is also immune mediated.

**Figure 3.**
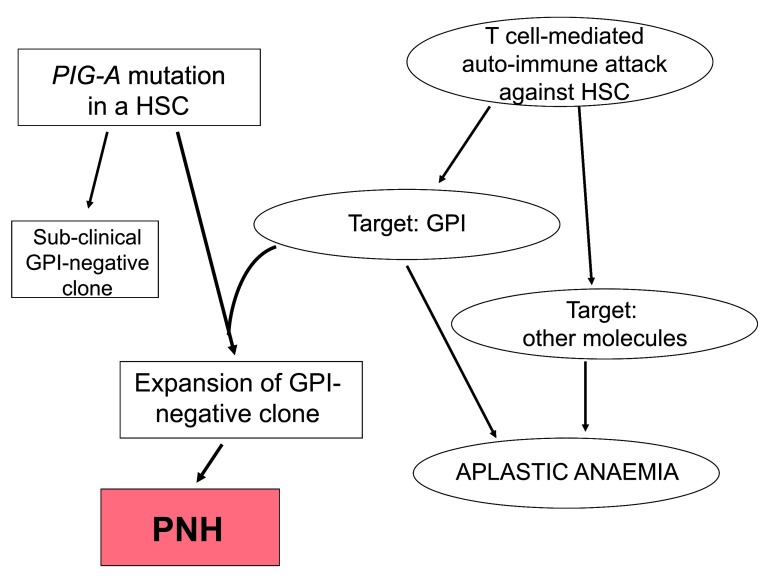
The dual pathogenesis of paroxysmal nocturnal hemoglobinuria (PNH). The diagram shows, on the right-hand side, the presumed pathogenesis of aplastic anemia (AA): a T-cell-mediated autoimmune attack damages hematopoietic stem cells (HSCs). The target of the auto-reactive T cells may be the glycosylphosphatidylinositol (GPI) molecule or another molecule expressed on HSCs. On the left-hand side, an inactivating mutation of the *PIG-A* gene in a HSC produces a GPI-negative hematopoietic clone. In the absence of the *PIG-A* mutant clone, the autoimmune attack, even if GPI is targeted, produces AA. In the absence of the autoimmune attack, a *PIG-A* mutant clone will be of no consequence (subclinical). Only if both a *PIG-A* mutant clone and a GPI-targeted autoimmune attack co-exist will the mutant clone expand and cause clinical PNH.

In principle, the target of immune selection might be ** either (i) a GPI-linked protein – peptides from which would be presented by the major histocompatibility complex (MHC) – or (ii) the GPI molecule itself (see Figure 3), which would be presented not by the MHC but by the structurally related molecule CD1d ^36^. The former possibility was not supported by the finding that the amino acid sequence of the CD3 of the βeta chain of the T cell receptor of selected CD57+ T cells was identical in PNH patients who had different HLA types ^37^. On the other hand, when CD8+ T cells were co-cultured with HLA-negative antigen-presenting B cells previously engineered to express CD1d, significantly higher levels of reactive T cells (producing interferon gamma were found in PNH patients compared to normal controls ^38^. When the antigen-presenting cells were further engineered to lose competence for endogenous synthesis of GPI, this reactivity became strictly dependent on loading their surface CD1d with exogenous human GPI produced by organic synthesis ^39^. Similar results were obtained with autologous antigen-presenting cells from peripheral blood monocytes from the same patients. These data provide the first direct evidence for the presence of GPI-reactive CD1d-restricted T cells in PNH patients ^38^.
